# Optimization of Extraction Conditions for Maximal Phenolic, Flavonoid and Antioxidant Activity from *Melaleuca bracteata* Leaves Using the Response Surface Methodology

**DOI:** 10.1371/journal.pone.0162139

**Published:** 2016-09-09

**Authors:** Wencheng Hou, Wei Zhang, Guode Chen, Yanping Luo

**Affiliations:** 1 Key Laboratory of Protection and Development Utilization of Tropical Crop Germplasm Resources, Ministry of Education; College of Environment and Plant Protection, Hainan University, Haikou, Hainan, 570228, P. R. China; 2 Hainan Provincial Forestry Science Institute, Tongshen Branch Offices, Wuzhishan, Hainan 572200, P. R. China; Islamic Azad University Mashhad Branch, ISLAMIC REPUBLIC OF IRAN

## Abstract

*Melaleuca bracteata* is a yellow-leaved tree belonging to the *Melaleuca* genus. Species from this genus are known to be good sources of natural antioxidants, for example, the “tea tree oil” derived from *M*. *alternifolia* is used in food processing to extend the shelf life of products. In order to determine whether *M*. *bracteata* contains novel natural antioxidants, the components of *M*. *bracteata* ethanol extracts were analyzed by gas chromatography–mass spectrometry. Total phenolic and flavonoid contents were extracted and the antioxidant activities of the extracts evaluated. Single-factor experiments, central composite rotatable design (CCRD) and response surface methodology (RSM) were used to optimize the extraction conditions for total phenolic content (TPC) and total flavonoid content (TFC). Ferric reducing power (FRP) and 1,1-Diphenyl-2-picrylhydrazyl radical (DPPH·) scavenging capacity were used as the evaluation indices of antioxidant activity. The results showed that the main components of *M*. *bracteata* ethanol extracts are methyl eugenol (86.86%) and *trans*-cinnamic acid methyl ester (6.41%). The single-factor experiments revealed that the ethanol concentration is the key factor determining the TPC, TFC, FRP and DPPH·scavenging capacity. RSM results indicated that the optimal condition of all four evaluation indices was achieved by extracting for 3.65 days at 53.26°C in 34.81% ethanol. Under these conditions, the TPC, TFC, FRP and DPPH·scavenging capacity reached values of 88.6 ± 1.3 mg GAE/g DW, 19.4 ± 0.2 mg RE/g DW, 2.37 ± 0.01 mM Fe^2+^/g DW and 86.0 ± 0.3%, respectively, which were higher than those of the positive control, methyl eugenol (FRP 0.97 ± 0.02 mM, DPPH·scavenging capacity 58.6 ± 0.7%) at comparable concentrations. Therefore, the extracts of *M*. *bracteata* leaves have higher antioxidant activity, which did not only attributed to the methyl eugenol. Further research could lead to the development of a potent new natural antioxidant.

## Introduction

Free radicals are produced during metabolism and can cause oxidative damage to cellular components including DNA, RNA, globular protein [1], proteases and unsaturated fatty acids [2]. Oxidative damage has been indicated as a primary cause of several diseases such as Alzheimer’s disease [3], hepatitis, cirrhosis and liver cancer [4], the innate immune system [5]. Antioxidants have been shown to eliminate or reduce the amount of free radicals and to decrease the incidence of these diseases [6, 7]. For example, antioxidant-rich extracts from *Salvia miltiorrhiza* leaves are used to treat liver cirrhosis by increasing Superoxide dismutase activity, scavenging free radicals and decreasing lipid peroxidation [8]. Antioxidants therefore play an important role in human life, but since some synthetic antioxidants can also have harmful side effects, natural substances like the extracts from *S*. *miltiorrhiza* leaves are often the preferred source [9].

The *Melaleuca bracteata* L. tree (S1 Fig family: *Myrtaceae*; genus: *Melaleuca*) has yellow leaves and is mainly distributed in tropical, subtropical and temperate regions [10]. Many studies on the cultivation and components of *M*. *bracteata* have reported that this plant has good temperature, light, moisture and salt tolerances [11–14] and that it can be cultured using the rapid propagation, cuttage and tissue culture techniques [15, 16]. Multiple studies have reported that methyl eugenol is the main component of *M*. *bracteata* leaves from Kenya and two locations in China, with concentrations up to 75%, 85.35% and 95.45%, respectively [17–19]. The next most abundant compounds in *M*. *bracteata* leaves are methyl cinnamate and carotene [20].

Plants of the *Melaleuca* genus have been used in food, pharmaceutical and cosmetic applications because of their high antioxidant activity [21–23]. Yoshimura *et al*. [24] isolated C-glycoside tannins from *M*. *squarrosa* with higher 1,1-Diphenyl-2-picrylhydrazyl radical (DPPH·) scavenging capacity than other tannins and flavonoids. Kim *et al*. [25] showed that the tea tree oil of *M*. *alternifolia* has good antioxidant activity and subsequently optimized a microemulsion model for its use. High DPPH· scavenging capacity has also been reported for the extracts of *M*. *armillaris* and *M*. *diosmifolia* (81.7 ± 1.1% and 90.6 ± 3.9%, respectively) [26–27]. Because *M*. *bracteata* belongs the *Melaleuca* genus but not much is known about its antioxidant activity, we set out to study its extracts in the hope of finding a new natural antioxidant.

“Full factorial design” and “Orthogonal experiment design” are used in many research areas to optimize experiments, but these methods are more complex and difficult to carry out [28]. Therefore, another method, Response surface methodology (RSM) [29], is widely used to design experiments, built models, express the response values, evaluate the effect of multiple factors, and show optimum conditions. With RSM, the relationships of several factors can be reflected with limited data, and intuitive models can be built, which will help us to get optimal results quickly.

In this study, we analyzed the components of ethanol extracts from *M*. *bracteata* leaves by Gas Chromatography-Mass Spectrometry (GC–MS). We then optimized three extraction factors (ethanol concentration, extraction time and extraction temperature) for *M*. *bracteata* leaf extraction by central composite rotatable design (CCRD) and RSM and investigated the feasibility of the obtained optimal extraction process.

## Materials and Methods

### Reagents

The following analytical grade or chemically pure reagents were purchased from commercial sources for this study: 30% hydrogen peroxide (H_2_O_2_), sodium acetate trihydrate (CH_3_CO_2_Na·3H_2_O), ferrous sulfate heptahydrate (FeSO_4_·7H_2_O), sodium nitrite (NaNO_2_), disodium hydrogen phosphate dodecahydrate (Na_2_HPO_4_·12H_2_O), sodium dihydrogen phosphate dihydrate (NaH_2_PO_4_·2H_2_O), ferric chloride hexahydrate (FeCl_3_·6H_2_O), anhydrous sodium carbonate (Na_2_CO_3_), potassium acetate (CH_3_CO_2_K), aluminum chloride (AlCl_3_). Potassium ferricyanide (K_3_[Fe(CN)_6_]), salicylic acid sodium, gallic acid monohydrate, Folin-ciocalteu reagent, 2,6-di-*tert*-butylphenol, Rutin (C_27_H_30_O_16_), *t*-butyl terephthalate phenol, Tris buffer, 2,4,6-Tri(2-pyridyl)-1,3,5-triazine (TPTA) and 2,2-diphenyl-1-picrylhydrazyl (DPPH) were obtained from Sigma chemicals.

### Preparation of extracts

Leaves of *M*. *bracteata*, normal cultivated plant, were collected in Wuzhishan city, Hainan province, China (N 109° 32′ 28″, E 18° 46′ 10″) when it was pruned (S1 File). The leaf samples were rinsed with deionized water, air-dried, pulverized, sieved through a 40 mesh sieve and stored in sealed containers until use.

The extracts were prepared following Gliwa’s method [30] under controlled temperature, time and ethanol concentration conditions from 5 g of dried *M*. *bracteata* leaf powder. The ratio of material to solvent was 1:20. The primary extract was filtered under reduced pressure, and the resulting filtrate was diluted to 200 mL in 50% ethanol to yield mother liquor. In order to measure sample absorbance, the mother liquor was further diluted 30-fold to yield the sample solution.

### Gas Chromatography-Mass Spectrometry (GC–MS) analysis

The sample for GC-MS was extracted in 50% ethanol at 50°C for 3 days. The extract was concentrated under reduced pressure to a constant weight and then diluted 10-fold with HPLC-grade *n*-hexane. The sample was analyzed on an Agilent 7890B-7000B GC–MS with the following conditions: injection volume: 1 μL; column: Hp-5ms (30 mm × 0.25 mm × 0.25 μm); temperature of sample inlet: 250°C; column temperature control: 60°C for 2 min, then heated to 300°C at a rate of 6°C/min and maintained at 300°C for 10 min; auxiliary heater temperature: 280°C; detection: 20–450 nm full scan; electron energy: −70 eV; and ion source temperature: 250°C. Components were identified by comparing the GC-MS results to mass spectral values obtained from the mass database NIST.

### Determination of Total Phenolic Content (TPC)

The TPC of the extracts was measured according to the method reported by Ibrahim *et al*. [31] with slight modifications. In brief, 1 mL of the sample solution was added to 0.5 mL Folin-Ciocalteu reagent and 5 mL ultrapure water and incubated at room temperature (RT) for 5 min. Then, 1 mL Na_2_CO_3_ (5% w/v) solution was added and incubated for 60 min at RT in the dark. The absorbance of the above mixture was measured at 760 nm using UV spectrophotometry. Ethanol (50%) was used as negative control and gallic acid as positive control. The TPC of the sample was compared to a gallic acid standard curve (y = 0.0093x + 0.0120; R^2^ = 0.9991, 1–96 μg/mL) and expressed relative to the equivalent standard concentrations (mg GAE/g DW, expressed as TPC per g powder). All determinations were performed in triplicate.

### Determination of Total Flavonoid Content (TFC)

The TFC of the extracts was measured as previously described [32] with slight modifications. In brief, 0.1 mL of a 10% (w/v) AlCl_3_ aqueous solution, 0.1 mL of a CH_3_CO_2_K solution (1 M) and 4.3 mL ultrapure water were sequentially added to 0.5 mL of the sample solution and incubated for 30 min at RT. The absorbance of the mixture was measured at 415 nm. Ethanol (50%) was used as negative control and Rutin as positive control. The TFC of the mixture was compared to the Rutin standard curve (y = 2.9700x + 0.0200; R^2^ = 0.9996, 0.01–0.64 mg/mL) and expressed relative to the equivalent standard concentrations (mg RE/g DW, expressed as TFC mass per g powder). All determinations were performed in triplicate.

### Determination of Ferric Reducing Power (FRP)

The FRP of the extract was assayed as previously described [33] with slight modifications. In brief, 0.1 mL of the sample solution was mixed thoroughly with 3 mL FRP solution [25 mL vinegar formate buffer solution (pH 3.6), 7.81 mg TPTA dissolved in 2.5 mL HCl (40 mM), and 2.5 mL FeCl_3_·6H_2_O (20 mM)] and incubated for 5 min at 37°C. The absorbance was measured at 593 nm. Ethanol (50%) was used as negative control and FeSO_4_·7H_2_O as positive control. The FRP of the sample was evaluated relative to the FeSO_4_·7H_2_O standard curve (y = 1.9890x + 0.0481; R^2^ = 0.9997, 8–768 μg/mL), expressed as FRP of the sample (mM Fe^2 +^/g DW).

### Determination of DPPH· scavenging capacity

The DPPH· scavenging capacity was measured as previously described [34–35] with slight modifications. In brief, the sample (0.5 mL) was added into 3.5 mL DPPH· solution (0.2 mM DDPH· solution diluted in 95% ethanol) and incubated at RT for 30 min. The sample absorbance (A_S_), ethanol absorbance (A_E_) or water absorbance (A_W_) of the sample, 95% ethanol or distilled water, respectively, were measured at 517 nm. The DPPH· scavenging capacity was calculated using the following formula:

$$\text{Scavenging capacity(\%)=}\left(1-\frac{A_{S}-A_{E}}{A_{W}}\right)\times 100\%$$

### Central composite rotatable design (CCRD) and response surface method (RSM)

Three factors and five levels of the central composite rotatable design (CCRD) method were used to optimize the extraction process. The three factors were ethanol concentration, extraction time and extraction temperature. Three center levels were decided by single-factor experiments, and the other two axis levels were based on an axis distance of ±1.68. All 18 entries were listed in Table 1. The empirical quadratic polynomial model was established by multiple linear regression analysis using the following formula:

$$RF=\beta_{\text{0}}\text{+}\overset{k}{\underset{i=1}{\sum }}\beta_{i}X_{i}+\overset{k}{\underset{i=1}{\sum }}\beta_{ii}X_{i}^{2}+\sum \underset{i<j}{\sum }\beta_{ij}X_{i}X_{j}$$

where *RF* is the response function; *β*_0_ is a constant term; *β*_i_, *β*_ii_, and *β*_ij_ are the regression coefficients of linear terms, quadratic terms and interaction effects, respectively; *X*_*i*_, *X*_*i*_^*2*^, and *X*_*i*_*X*_*j*_ are the linear, quadratic and interaction effects, respectively; and k is the number of processing elements, where i < j.

**Table 1:** Central composite rotatable design and four response variables for optimization of the extraction process.

No.	Process variables‒real/coded values			Responses ^a^			
	X_1_. Time(d)	X_2_. *T*(°C)	X_3_. EtOH (%)	TPC (mg GAE/g DW)	TFC (mg RE/g DW)	FRP (mM Fe^2+^/g DW)	DPPH· scavenging capacity (%)
1	4 (1)	60 (1)	60 (1)	99.4±0.2	10.2±0.3	1.80±0.07	59.6±1.4
2	4 (1)	60 (1)	20 (−1)	87.7±0.3	14.7±0.4	1.99±0.01	86.7±1.1
3	4 (1)	40 (−1)	60 (1)	90.6±0.2	8.6±0.2	1.77±0.01	78.1±1.3
4	4 (1)	40 (−1)	20 (−1)	72.1±1.3	14.6±0.2	1.95±0.03	80.3±1.4
5	2 (−1)	60 (1)	60 (1)	100.1±0.4	9.9±0.2	1.65±0.06	63.5±1.4
6	2 (−1)	60 (1)	20 (−1)	81.5±1.1	17.9±0.3	2.01±0.03	88.5±0.7
7	2 (−1)	40 (−1)	60 (1)	88.2±0.2	9.4±0.2	1.52±0.04	87.7±0.9
8	2 (−1)	40 (−1)	20 (−1)	65.1±1.4	19.1±0.3	1.83±0.04	88.6±1.0
9	4.68 (1.68)	50 (0)	40 (0)	88.4±0.7	14.2±0.2	2.08±0.02	71.2±0.8
10	1.32 (−1.68)	50 (0)	40 (0)	84.9±1.1	16.1±0.2	1.96±0.04	84.8±1.2
11	3 (0)	66.81 (1.68)	40 (0)	86.9±0.5	16.0±0.1	2.10±0.05	78.7±1.4
12	3 (0)	33.18 (−1.68)	40 (0)	66.7±0.6	14.3±0.4	2.07±0.03	94.2±0.9
13	3 (0)	50 (0)	73.64 (1.68)	104.4±0.4	5.5±0.3	1.20±0.01	60.1±1.2
14	3 (0)	50 (0)	6.36 (−1.68)	75.0±1.3	15.7±0.6	1.68±0.01	86.5±0.9
15	3 (0)	50 (0)	40 (0)	86.9±1.0	20.2±0.3	2.41±0.06	89.9±0.2
16	3 (0)	50 (0)	40 (0)	88.7±1.6	20.1±0.1	2.36±0.03	89.8±0.3
17	3 (0)	50 (0)	40 (0)	87.7±1.2	20.2±0.1	2.39±0.04	88.7±0.8
18	3 (0)	50 (0)	40 (0)	88.7±1.1	20.7±0.2	2.40±0.03	90.1±0.2

^a^ Responses are the means ± SD (n = 3)

### Statistical Analysis

SPSS 22.0 software was used to analyze the data by ANOVA (p <0.05). In the response surface experiment, Design-Expert 8.0.6 analysis software was used to analyze the data and draw the response surface plots.

## Results and Discussion

### GC–MS analysis

**Fig 1:**
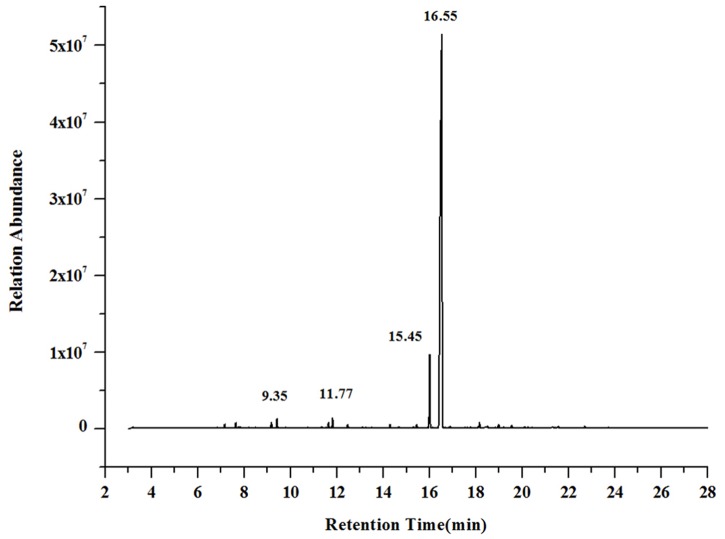
GC-MS spectrometry of *M*. *bracteata* leaves ethanol extract.

The composition of the *M*. *bracteata* leaf ethanol extract was analyzed using GC–MS, and the total ion flow chart is depicted in Fig 1. Each peak of the ion flow chart was scanned by mass spectrometry and compared to the NIST98 mass spectrum database to identify 36 unique compounds in the leaf extract. The percent contribution of each compound in the extract was calculated using the peak area normalization method (Table 2).

**Table 2:** The components of *M*. *bracteata* leaves ethanol extract.

No.	R.Time(min)	Compound	Formula	Molecular mass	Content (%)
1	6.829	*β*-Pinene	C_10_H_16_	136.23	0.05
2	7.167	*α*-Phellandrene	C_10_H_16_	136.23	0.27
3	7.64	*o*-Cymene	C_10_H_14_	134.22	0.40
4	7.741	*D*-Limonene	C_10_H_16_	136.23	0.10
5	7.818	Eucalyptol	C_10_H_18_O	154.24	0.09
6	8.18	*α*- Pinene	C_10_H_16_	136.23	0.05
7	8.458	*γ*-Terpinene	C_10_H_16_	136.23	0.03
8	9.178	Isoterpinolene	C_10_H_16_	136.23	0.49
9	9.418	Linalool	C_10_H_18_O	154.24	0.73
10	9.763	Phenylethyl Alcohol	C_8_H_10_O	122.16	0.03
11	10.709	Citronellal	C_10_H_18_O	154.24	0.03
12	11.332	Terpinen-4-ol	C_10_H_18_O	54.24	0.11
13	11.499	2-(4-Methylphenyl)propan-2-ol	C_10_H_14_O	150.21	0.05
14	11.642	*α*-terpineol	C_10_H_18_O	54.24	0.47
15	11.819	Estragole	C_10_H_12_O	148.22	0.81
16	12.47	Citronellol	C_10_H_20_O	156.26	0.30
17	13.232	Citronellyl formate	C_11_H_20_O_2_	172.25	0.03
18	14.293	Cis-Methyl cinnamate	C_10_H_10_O_2_	162.17	0.32
19	14.673	Trans- Methyl geranate	C_11_H_18_O_2_	182.25	0.10
20	15.309	3,7-dimethyloct-6-en-1-yl 4-methylpentanoate	C_16_H_30_O_2_	254.40	0.01
21	15.452	Eugenol	C_10_H_12_O_2_	164.19	0.31
22	16.023	*trans*-Methyl cinnamate	C_10_H_10_O_2_	162.18	6.41
23	16.548	Methyleugenol	C_11_H_14_O_2_	178.22	86.86
24	16.889	4,8,8-trimethyl-2-methylene-4-vinylbicyclo[5.2.0]nonane	C_15_H_21_	201.32	0.13
25	17.53	(1*R*,4*R*,4a*R*)-1-isopropyl-4,7-dimethyl-1,2,3,4,4a,5-hexahydronaphthalene	C_15_H_24_	204.35	0.03
26	17.61	(1*Z*,4*Z*,7*Z*)-1,5,9,9-tetramethylcycloundeca-1,4,7-triene	C_15_H_24_	204.35	0.05
27	18.17	*cis*-*b*-Copaene	C_15_H_24_	204.35	0.49
28	18.483	Isolepidozene	C_15_H_24_	204.35	0.09
29	18.991	*cis*-Calamenene	C_15_H_22_	202.34	0.30
30	19.179	4-isopropyl-1,6-dimethyl-1,2,3,4,4a,7-hexahydronaphthalene	C_15_H_24_	204.35	0.05
31	19.551	Elemicin	C_12_H_16_O_3_	254.35	0.20
32	20.101	*E*-spatulenol	C_15_H_24_O	220.35	0.14
33	20.39	(-)-Globulol	C_15_H_26_O	222.36	0.04
34	21.309	*T*-Muurolol	C_15_H_26_O	222.37	0.08
35	21.545	*α*- Cadinol	C_15_H_26_O	222.37	0.11
36	22.701	Trimethyl gallic acid methyl ester	C_11_H_14_O_5_	226.20	0.16

Thirty-nine peaks appeared in the GC–MS spectra, and 36 compounds were identified, accounting for 99.2% of the total content. The main classes of compounds were phenols, terpenes and esters. The content of methyl eugenol was highest, making up about 86.86% of the total content, followed by *trans*-cinnamic acid methyl ester, accounting for 6.41% of the content. Estragole and linalool made up 0.81% and 0.73%, respectively. Methyl eugenol has been applied in many ways, for example as growth inhibitor against *Plasmodium falciparum* and parasitic mites [36] or as repellent against *Tribolium castaneum* [37]. Therefore, *M*. *bracteata* likely has practical value because of its high methyl eugenol content. Furthermore, because phenols can have high antioxidant activity [38–39], the ethanol extract of *M*. *bracteata* leaves might also have potent antioxidant activity.

### Single-factor extraction optimization

**Fig 2:**
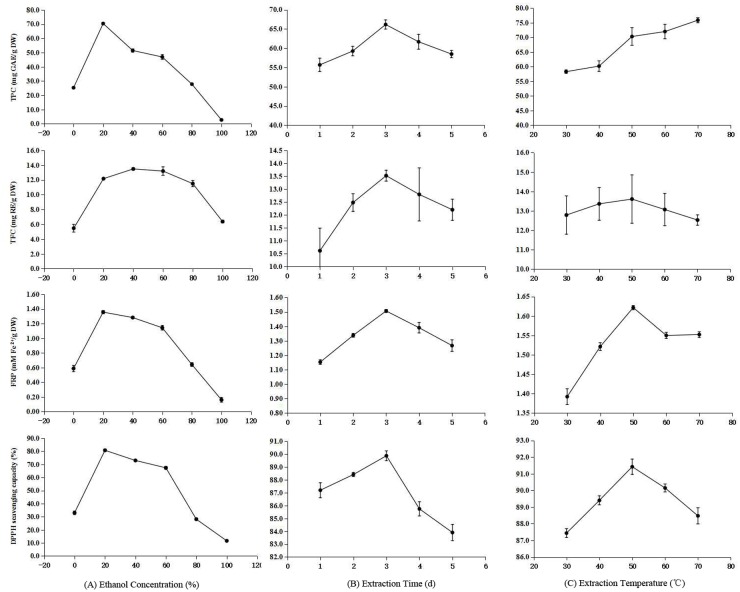
Effects of concentration, time and temperature on TPC, TFC, FRP and DPPH· scavenging capacity. (A) ethanol concentration (*T* = 50°C, Time = 3 d), (B) Extraction time (Ethanol = 40%, T = 50°C), (C) Extraction temperature (Ethanol = 40%, Time = 3 d).

First, the contributions of the ethanol concentration, extraction time and extraction temperature to the TPC, TFC, FRP and DPPH· scavenging capacity of the resulting extract were determined by keeping two of the three extraction factors constant and varying the third (Fig 2).

#### Effects of ethanol concentration

Fig 2A shows that for a 3-day extraction at 50°C, the TPC, TFC, FRP and DPPH· scavenging capacity first increase and then decrease with increasing ethanol concentrations. The highest TPC (70.4 mg) was obtained at 20% ethanol, and the highest TFC (13.5 mg) at 40% ethanol. FRP and DPPH• scavenging capacity followed a similar trend to TPC and peaked at 20% ethanol with 1.36 mM and 80.7%, respectively. This is in accordance with Silva *et al*. [40] who reported a reduced antioxidant activity of the *Inga edulisa* extract at higher concentrations of organic solvent. Singh *et al*. [41] also reported that the extraction of flavonoids correlated well with the ethanol concentration.

#### Effects of extraction time

Mokrani and Madani [42] reported that the extraction time significantly affects the antioxidant activity of the extract. Our results showed that the TPC, TFC, FRP and DPPH• scavenging capacity first increased and then decreased with longer extraction times at 40% ethanol and 50°C (Fig 2B). The optimal extraction time for all four evaluation indices was 3 days, with corresponding TPC, TFC, FRP and DPPH• scavenging capacity values of 66.2±1.2 mg, 13.5±0.2 mg, 1.50 mM and 90.6%, respectively. Interestingly, the DPPH• scavenging capacity increased only modestly from 1 to 3 days but decreased drastically for more than 3 days. The reason was that, on the one hand, the content of the TPC, TFC was decrease for more than 3 days, on the other hand, possibly because some substances in the leaves underwent spontaneous oxidation with prolonged incubation [43–44].

#### Effects of extraction temperature

During a 3-day extraction in 40% ethanol, the TFC, FRP and DPPH• scavenging capacity responses to increasing incubation temperatures followed a trend similar to the one observed in the other two single-factor experiments, peaking at 50°C (Fig 2C). When the temperature was more than 60°C, the scavenging capacity of extracts was decreased, although, the TPC continued to rise as the temperature increased for the entire tested temperature range. This could have been due to more substances dissolving, hydrolyzing reducing sugars, or other redox reactions occurring at higher temperatures, resulting in increasing TPCs [45–46]. Because of redox reactions itself, the scavenging capacity was not increased, but decreased.

In conclusion, ethanol concentrations (20%, 40% and 60%), extraction time (2-, 3- and 4-day) and extraction temperatures (40°C, 50°C and 60°C) were selected as the three levels for the RSM experiment.

### Model establishment

Based on the results of the single-factor experiments (Fig 2), the levels of the three factors were determined (ethanol concentrations: 20%, 40% and 60%; extraction times: 2, 3 and 4 days; extraction temperatures: 40°C, 50°C and 60°C), and the other two axis levels were based on an appropriate axis distance ±1.68. The parameters and resulting responses are shown in Table 1.

The values of the four evaluation indices for each extracting condition are listed in Table 1. The maximal TPC was 104.4 ± 0.4 mg and obtained in a 3-day extraction at 50°C in 73.64% ethanol (No. 13). The maximal TFC (20.7 ± 0.2 mg) and RFP (2.41 ± 0.06 mM) were obtained in a 3-day extraction at 50°C in 40% ethanol (No. 18 and No. 15, respectively). The DPPH• scavenging capacity had the highest value of 94.2 ± 0.9% in a 3-day extraction at 33.18°C in 40% ethanol (No. 12). From the multiple linear regression analyses of the 18 data entries, empirical second-order polynomial models of TPC, TFC, FRP and DPPH• scavenging capacity were derived (Table 3). therein, the insignificant parameters in models with the t-test (*P* > 0.05) were deleted.

**Table 3:** Empiric second-order polynomial model of TPC, TFC, FRP and DPPH• scavenging capacity.

Response	Model equations	Probability of lack of fit	*R*^2^
TPC(mg GAE/g DW)	Y = -0.1950+0.3040*X*_*1*_+0.2625*X*_*2*_+0.0530*X*_*3*_-4.1746×10^-3^*X*_*1*_*X*_*3*_-4.7992×10^-4^*X*_*2*_*X*_*3*_-8.5865×10^-3^*X*_*1*_^2^-2.0785×10^-3^*X*_*2*_^2^+9.4163×10^-5^*X*_*3*_^2^	0.1885	0.9846 ^b^
TFC(mg RE/g DW)	Y = -2.0165+0.7998*X*_*1*_+0.2014*X*_*2*_+0.0413*X*_*3*_+4.1658×10^-3^*X*_*1*_*X*_*2*_+5.3756×10^-3^*X*_*1*_*X*_*3*_+2.9257×10^-4^*X*_*2*_*X*_*3*_-0.2215*X*_*1*_^2^-2.2132×10^-3^*X*_*2*_^2^-1.2047×10^-3^*X*_*3*_^2^	0.1533	0.9939 ^b^
FRP(mM Fe^2+^/g DW)	Y = -0.3080+0.3064*X*_*1*_+0.0398*X*_*2*_+0.0197*X*_*3*_-1.1392×10^-3^*X*_*1*_*X*_*2*_+7.3794×10^-4^*X*_*1*_*X*_*3*_-0.0433*X*_*1*_^2^-3.5213×10^-4^*X*_*2*_^2^-3.0737×10^-4^*X*_*3*_^2^	0.0926	0.9862 ^b^
DPPH• scavenging capacity (%)	Y = 5.0992+0.7392*X*_*1*_+0.0783*X*_*2*_+0.1333*X*_*3*_+7.9299×10^-3^*X*_*1*_*X*_*2*_-1.7865×10^-3^*X*_*2*_*X*_*3*_-0.2213*X*_*1*_^2^-5.6805×10^-4^*X*_*2*_^2^-8.1554×10^-4^*X*_*3*_^2^	0.0933	0.9886 ^b^

X_1_ = Extraction Time (d); X_2_ = Extraction Temperature (°C); X_3_ = EtOH (%).

^b^: means p < 0.001

As shown in Table 3, the probabilities of lack-of-fit of the four models were not significant but the correlation coefficients were significant with an F-test. These results indicated that the empirical second-order polynomial models were suitable for the experimental data and that the response surface analysis can be applied to optimize the extraction of the antioxidant substances from *M*. *bracteata* leaves and to evaluate the antioxidant activity of the extract.

### Correlation analysis

The correlations between any two of the four evaluation indices under different factors are shown in Table 4. Under the ethanol concentration, the correlations of TPC and FRP, TPC and DPPH• scavenging capacity, and FRP and DPPH• scavenging capacity were significant (*P* <0.005). The correlation of TFC and FRP was also significant (*P* <0.05) but the correlations of TFC and TPC and TFC and DPPH• scavenging capacity were not (*P* >0.05). This suggests that both the ethanol concentration and TPC correlate with the antioxidant activity of the extract, and that the FRP and DPPH• scavenging capacity can be selected as the antioxidant activity index. Our results were similar to those previously reported [47]. On the other hand, the main component of leaf extracts identified by GC-MS was methyl eugenol, suggesting that the phenolic compound likely plays a major role in the antioxidant activity of the extract. Because of their relatively low concentration, flavonoids are less likely to significantly contribute to the antioxidant activity of the extract.

**Table 4:** Correlation between different indicators under each factor.

*r*^*2*^	EtOH (%)			Time (d)			*T* (°C)		
	TPC	TFC	FRP	TPC	TFC	FRP	TPC	TFC	FRP
TFC	0.576^NS^			0.844^c^			0.051^NS^		
FRP	0.952^a^	0.683^c^		0.958^c^	0.939^b^		0.517^NS^	0.228^NS^	
DPPH•	0.942^a^	0.599^NS^	0.975^a^	0.225^NS^	0.094^NS^	0.236^NS^	0.174^NS^	0.623^NS^	0.782^c^

^a^ p < 0.005,

^b^ p < 0.01,

^c^ p < 0.05,

NS: non-significant; r: correlation coefficient

Under the extraction time, the correlations among the TFC, TPC and FRP, but not the DPPH• scavenging capacity with TFC and TPC, were significant. This suggests that the extraction time has the same effect on the TPC, TFC and FRP but is not a significant factor for the DPPH• scavenging capacity. The correlations between evaluation indices were not significant under the extraction temperature except for FRP and DPPH• scavenging capacity (*r*^2^ = 0.782, *P* <0.05), suggesting that the extraction temperature has complex effects on the extracts.

The results of the correlation analyses indicate that ethanol concentration is the biggest factor determining the composition of *M*. *bracteata* leaf extracts, followed by the extraction time. Matching the above correlation analyses, the concentration of ethanol was directly related to the TPC of extracts which determined the antioxidant activity of the samples.

### RSM analysis

**Fig 3:**
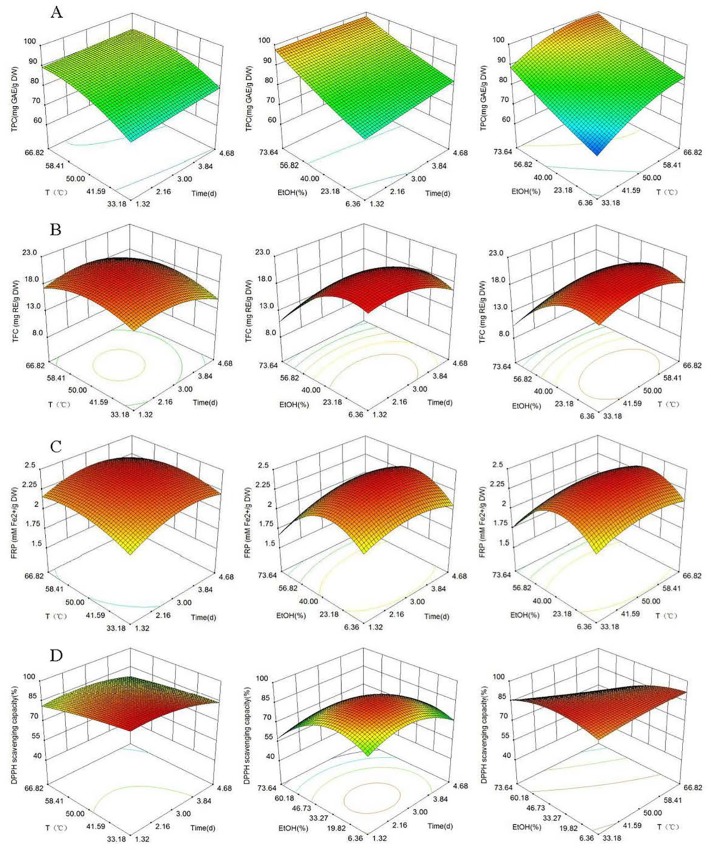
Response surface of TPC, TFC, FRP and DPPH• scavenging capacity. (A)TPC, (B)TFC, (C)FRP and (D)DPPH• scavenging capacity. Color gradients indicate the level of optimization (red = high, green = intermediate, blue = low).

Based on the empirical second-order polynomial model, the experimental data was analyzed by RSM using the Design-Expert 8.0.6 software (Fig 3). The X- and Y-axes of the three-dimensional response surfaces represent two factors, for example ethanol concentration and extraction time (extraction temperature = 50°C), ethanol concentration and extraction temperature (extraction time = 3 days), or extraction time and extraction temperature (ethanol concentration = 40%). The Z-axes represent one of the four evaluation indices (TPC, TFC, FRP or DPPH• scavenging capacity). Three-dimensional response surfaces were constructed as depicted in Fig 3.

The evaluation index is associated with the slope of the response surface, i.e., the bigger the slope, the more quickly the evaluation index increases. In addition, the interaction of two factors is reflected in the contour of the plot, so that a rounded contour line indicates a weak interaction of two factors and a distorted contour indicates a significant interaction of two factors [48]. Fig 3A shows that the slope of the response surface follows an upward trend with two factors increasing. This suggested that the three factors had a significant impact on the TPC. We speculate that with increasing extraction temperatures, extraction times or ethanol concentrations, more polyphenols were dissolved and then hydrolyzed to increase the TPC within the tested scope.

Fig 3B–3D shows that the evaluation indices for three interaction factors follow similar trends, with the peak value first appearing and then decreasing as the two factors increase. The contour plot was almost circular in Fig 3B, indicating that the extraction time and temperature did not affect the TFC significantly while the interactions between other factors had significant effects.

### Verification of optimal experimental conditions

Based on the empirical second-order polynomial model and RSM, the optimal extraction conditions of each of the four evaluation indices are listed in Table 5. Under optimal conditions, maximal values of TPC (98.7 mg), TFC (21.6 mg), FRP (2.40 mM) and DPPH• scavenging capacity (94.7%) were obtained and differed only minimally from the predicted values, indicating that the established model was effective.

**Table 5:** Experimental and predicted values of each evaluation index under optimal conditions.

Responses	Optimum extraction conditions			Maximum value	
	Time (d)	T (°C)	EtOH (%)	Experimental ^a^	Predicted
TPC(mg GAE/g DW)	2.83	56.17	60.00	98.7±1.2	99.5
TFC(mg RE/g DW)	2.63	49.89	29.06	21.6±0.3	21.7
FRP (mM Fe^2+^/g DW)	3.17	51.37	35.89	2.40±0.03	2.41
DPPH· scavenging capacity (%)	2.39	40.00	37.92	94.7±0.8	95.2

^a^ Responses are the means ± SD(n = 3)

Kuppusamy *et al*. [27] reported that the TPC of *M*. *disomifolia* extracts was 39.0 mg and thus much lower than that of *M*. *bracteata* (98.7 mg), but the TFC was 30.7 mg and thus a little bit higher than that of *M*. *bracteata* (21.6 mg). Comprehensive analyses determined that the DPPH• scavenging capacity of the *M*. *bracteata* extract is better than that of the *M*. *disomifolia* extract. Therefore, the *M*. *bracteata* leaves extract has excellent antioxidant activity compared to other plant extracts.

In order to determine the maximum value of the evaluation indices that can be achieved simultaneously under the same extraction conditions, an optimal extraction condition of 3.65 days at 53.26°C in 34.81% ethanol was first set based on the RSM (Table 6). Extraction under these conditions yielded TPC, TFC, FRP and DPPH• scavenging capacity of 88.6±1.3 mg, 19.4±0.2 mg, 2.37±0.01 mM and 86.0±0.3%, respectively. These experimental values did not differ significantly from the predicted values, further confirming the accuracy of our model. However, the measured values of each evaluation index extracted under the universal extraction conditions were lower than those obtained in the extractions optimized specifically for each individual evaluation index. The reason was that different extraction conditions were optimal for different evaluation indices, and the universal extraction conditions were not sufficient for all evaluation indices to reach their maximum values. Therefore, different extraction conditions were selected to obtain the maximum values according to experiment aims. For example, in order to obtain the highest DPPH• scavenging capacity, the sample was extracted for 2.39 days at 40°C in 37.92% ethanol.

**Table 6:** Experimental and predicted values of evaluation indices extracted under the same conditions.

Optimum Condition		TPC(mg GAE/g DW)	TFC(mg RE/ g DW)	FRP (mM Fe^2+^/g DW)	DPPH· scavenging capacity (%)
Time (3.65d) *T* (53.26°C) EtOH (34.81%)	Experimental^a^	88.6±1.3	19.4±0.2	2.37±0.01	86.0±0.3
	Predicted	88.8	19.5	2.38	86.3

^a^ Experiment are the means ± SD (n = 3)

Since the main component of the extracts identified by GC-MS is methyl eugenol, the FRP and DPPH• scavenging capacity of methyl eugenol was next investigated in order to determine whether methyl eugenol is the key component of the antioxidant activity of extracts. The concentration of methyl eugenol was prepared to match the one in *M*. *bracteata* leaf extracts (88.8 mg GAE/g DW). Our results showed that the FRP and DPPH• scavenging capacities of methyl eugenol were 0.97 ± 0.02 mM and 58.6 ± 0.7%, respectively, lower than in the whole extract. This implies that while methyl eugenol is one of the factors contributing to the antioxidant activity of *M*. *bracteata* leaf extracts, other components of the extracts have stronger antioxidant activity.

Through our experiments, the plant *M*. *bracteata* shows stronger antioxidant activity. There are three mechanisms of antioxidant activity about polyphenolic compounds in the literature [49–50]. The first one, polyphenolic compounds chelate metal iron to form catalytic activity center, the catalytic center played a important role in antioxidant reaction; the second, polyphenolic compounds are easily oxidized to quinines or ketones substances, provide the hydrogen ion, which combines DPPH• to change the OD value; the third, polyphenolic compounds can inhibit some antioxidant enzyme, and reduce the enzymatic activity. The antioxidant mechanism of *M*. *bracteata* leaves extract may be the second method, the same result has been reported by Kumarappan[51].

## Conclusions

Of the 36 different compounds identified by GC–MS in the aqueous ethanol extracts of *M*. *bracteata* leaves, methyl eugenol is the major component (86.86%). The extraction conditions of *M*. *bracteata* leaves were optimized by single-factor experiments and RSM. The three factors (time, temperature and ethanol concentration) strongly affected the content of the extraction, with the ethanol concentration being the most significant determinant. Optimal total antioxidant extraction under universal extraction conditions was predicted for a 3.65-day incubation at 53.26°C in 34.81% ethanol and yielded an extract with 88.6 ± 1.3 mg TPC, 19.4 ± 0.2 mg TFC, 2.37 ± 0.01 mM FRP and 86.0 ± 0.3% DPPH• scavenging capacity. When extraction conditions were optimized for each evaluation index individually, the TPC, TFC, FRP and DPPH• scavenging capacity reached peak values of 98.7 ± 1.2 mg, 21.6 ± 0.3 mg, 2.40 ± 0.03 mM and 94.7 ± 0.8%, respectively. Predicted values were verified experimentally, confirming the accuracy of the model generated from the analysis.

## Supporting Information

S1 Fig Melaleuca bracteata. (TIF)

S1 File Confirmation. (PDF)

S1 Table Raw data of absorbance of TPC, TFC, FRP and DPPH free scavenging capacity. (XLS)

S2 Table Data of GC-MS figure (Fig 1). (XLS)

S3 Table Raw data of absorbance in the single factor (Fig 2). (XLS)

S4 Table Raw data of absorbance(Table 5). (XLS)

S5 Table Raw data of absorbance(Table 6). (XLS)

## References

[1] Chamani J, Moosavi-Movahedi AA, Rajabi O, Gharanfoli M, Momen-Heravi M, Hakimelahi GH, et al Cooperative α-helix formation of β-lactoglobulin induced by sodium n-alkyl sulfates. Journal of colloid and interface science. 2006; 293(1), 52–60. 10.1016/j.jcis.2005.06.015 15996676

[2] Sivanandham V. Free radicals in health and diseases-a mini review. Pharmacology online 2011; 1: 1062–1077.

[3] Wojtunik-Kulesza KA, Oniszczuk A, Oniszczuk T, Waksmundzka-Hajnos M. The influence of common free radicals and antioxidants on development of Alzheimer’s Disease. Biomedicine & Pharmacotherapy. 2016; 78: 39–49. 10.1016/j.biopha.2015.12.024 26898423

[4] Hefnawy HTM, Ramadan MF. Protective effects of *Lactuca sative* ethanolic extract on carbon tetrachloride induced oxidative damage in rats. Asian Pacific Journal of Tropical Disease. 2013; 3(4): 277–285. 10.1016/S2222-1808(13)60070-5

[5] Asoodeh A, Zardini HZ, Chamani J. Identification and characterization of two novel antimicrobial peptides, temporin-Ra and temporin-Rb, from skin secretions of the marsh frog (Rana ridibunda). Journal of Peptide Science. 2012; 18(1), 10–16. 10.1002/psc.1409 21956830

[6] Shim JY, Kim MH, Kim HD, Ahn JY, Yun YS, Song JY. Protective action of the immunomodulator ginsan against carbon tetrachloride-induced liver injury via control of oxidative stress and the inflammatory response. Toxicology and Applied Pharmacology. 2010; 242(3): 318–325. 10.1016/j.taap.2009.11.005 19913046

[7] Qian ZJ, Jung WK, Kim SK. Free radical scavenging activity of a novel antioxidative peptide purified from hydrolysate of bullfrog skin, *Rana catesbeiana Shaw*. Bioresource Technology. 2008; 99(6): 1690–1698. 10.1016/j.biortech.2007.04.005 17512726

[8] Tao YY, Liu CH. Progress of research on mechanism of *salvia miltiorrhiza* and its chemical ingredients against liver fibrosis. Journal of Chinese Integrative Medicine. 2004; 2(2): 145–148. 10.3736/jcim20040223 15339483

[9] Sokmen M, Serkedjieva J, Daferera D, Gulluce M, Polissiou M, Tepe B, et al In vitro antioxidant, antimicrobial, and antiviral activities of the essential oil and various extracts from herbal parts and callus cultures of origanum acutidens. Journal of Agricultural and Food Chemistry. 2004; 52(11): 3309–3312. 10.1021/jf049859g 15161188

[10] Padalia RC, Verma RS, Chauhan A, Goswami P, Verma SK, Darokar MP. Chemical composition of *Melaleuca linarrifolia* Sm. from India: a potential source of 1,8-cineole. Industrial Crops and Products. 2015; 63: 264–268. 10.1016/j.indcrop.2014.09.039

[11] Liu GL, Zhou X, Pan YZ, Chen QB. Effects of low temperature stress on cold-resistance physiological characteristics of *Melaleuca bracteata* cv.'Revolution Gold'. Journal of Anhui Agricultural Sciences. 2010; 38(22): 12062–12064.

[12] Yang LQ, Wu Y, Song ML, Liu GL. Pigment content of *Melaleuca bracteata* ev. 'Revolution Gold' under different sun-shading intensity. Guizhou Agricultural Sciences. 2014; 42(7): 150–154.

[13] Hou ST, Zhang Q, Liu SQ, Zeng LY, Wu Y, Liu GL. Growth and physiological responses of *Melaleuca bracteata* cv. 'Revolution Gold' to water stress. Acta Botanica Boreali-Occidentalia Sinica. 2014; 34(12): 2491–2499.

[14] Ai XM, Yang P, Li Y, Fan GS. Variation of physiological indexes of *Melaleuca bracteata* seedlings under acid, alkali and salt stress. Journal of West China Forestry Science. 2014;43(1):29–33.

[15] Wu LJ, Weng QY, Chen BH, Gao N, Shen JN. Study on tissue culture and rapid propagation of *Melaleuca altennifolia* of high essential oil. Journal of Fujian College of Forestry. 2010; 30(4): 314–319.

[16] Qu FX. Preliminary studying factor on effects germination and calls inducing of *Melaleuca bracteata*. Northern Horticulture. 2011; 8: 152–154.

[17] Cosgrove DJ, Thain EM. Oil of *Melaleuca bracteata* from Kenya. Bull. Imp. Inst., 1948; 46: 46–50.

[18] Ye ZM, Liu X, Shen MJ, Chen JY, Chen XD, Li YY, et al Optimization of Process for Extraction from *Melaleuca bracteata* Essential Oil with Organic Solvent and Chemical Composition Identify. Chinese Journal of Tropical Crops. 2014; 35(5): 992–998.

[19] Zhong CY, Huang ZQ, Liang ZY, Chen MY. Study on Chemical Components of Essential Oil From the Branches and Leaves of *Melaleuca bracteata*. Flavour Fragrance Cosmetics. 2009; 6: 8–10.

[20] Lin XH, Chen SR. Parameter Studies of Process Optimization for Carotenoid Extraction from Revolution Gold. Chinese Journal of Tropical Crops. 2015; 36(5): 991–997.

[21] Budhiraja SS, Cullum ME, Sioutis SS, Evangelista L, Habanova ST. Biological activity of *Melaleuca alternifolia* (tea tree) oil component, terpinen-4-ol, in human myelocytic cell line HL-60. Journal of manipulative and physiological therapeutics. 1999; 22(7): 447–453. 10.1016/S0161-4754(99)70033-3 10519561

[22] Juergens UR, Dethlefsen U, Steinkamp G, Gillissen A, Repges R, Vetter H. Anti-inflammatory activity of 1.8-cineol (eucalyptol) in bronchial asthma: a double-blind placebo-controlled trial. Respiratory medicine. 2003; 97(3): 250–256. 10.1053/rmed.2003.1432 12645832

[23] Silva CJ, Barbosa LCA, Demuner AJ, Montanari RM, Pinheiro AL, Dias I, et al Chemical composition and antibacterial activities from the essential oils of Myrtaceae species planted in Brazil. Química Nova. 2010; 33(1): 104–108. 10.1590/S0100-40422010000100019

[24] Yoshimura M, Ito H, Miyashita K, Hatano T, Taniguchi S, Amakura Y, et al Flavonol glucuronides and C-glucosidic ellagitannins from *Melaleuca squarrosa*. Phytochemistry. 2008;69(18): 3062–3069. 10.1016/j.phytochem.2008.04.004 18501392

[25] Kim S, Ng WK, Shen S, Dong Y, Tan RBH. Phase behavior, microstructure transition, and antiradical activity of sucrose laurate/propylene glycol/the essential oil of Melaleuca alternifolia/water microemulsions. Colloids and Surfaces A: Physicochemical and Engineering Aspects. 2009; 384(1–3): 289–297. 10.1016/j.colsurfa.2009.07.043

[26] Kuppusamy S, Thavamani P, Megharaj M, Nirola R, Lee YB, Naidu R. Assessment of antioxidant activity, minerals, phenols and flavonoid contents of common plant/tree waste extracts. Industrial Crops and Products. 2016; 83: 630–634. 10.1016/j.indcrop.2015.12.060

[27] Kuppusamy S, Thavamani P, Megharaj M, Venkateswarlu K, Lee YB, Naidu R. Potential of *Melaleuca disomifolia* leaf as a low-cost adsorbent for hexavalent chromium removal from contaminated water bodies. Process Safety and Environmental Protection.2016; 100: 173–182.

[28] Tang JC, Gong GC, Su H, Wu FH, Herman C. Performance evaluation of a novel method of frost prevention and retardation for air source heat pumps using the orthogonal experiment design method. Applied Energy. 2016; 169: 696–708. 10.1016/j.apenergy.2016.02.042

[29] Kim SC. Application of response surface method as an experimental design to optimize coagulation–flocculation process for pre-treating paper wastewater. Journal of Industrial & Engineering Chemistry. 2016; 38(2): 93–102. 10.1016/j.jiec.2016.04.010

[30] Gliwa J, Gunenc A, Ames NP, Willmore WG, Hosseinian FS. Antioxidant activity of alkylresorcinols from rye bran and their protective effects on cell viability of PC-12 AC cells. Journal of agricultural and food chemistry. 2011; 59(21): 11473–11482. 10.1021/jf2023353 21910481

[31] Ibrahim MM, AL Sahli AAA, Alaraidh IA, Al-Homaidan AA, Mostafa EM, El-Gaaly GA. Assessment of antioxidant activities in roots of Miswak (Salvadora persica) plants grown at two different locations in Saudi Arabia. Saudi Journal of Biological Sciences. 2015; 22(2): 168–175. 10.1016/j.sjbs.2014.11.019 25737648 PMC4336451

[32] Chang CC, Yang MH, Wen HM, Chern JC. Estimation of total flavonoid content in propolis by two complementary colorimetric methods. Journal of Food and Drug Analysis. 2002;10(3): 178–182.

[33] Du B, Xu BJ. Oxygen radical absorbance capacity (ORAC) and ferric reducing antioxidant power (FRAP) of *β*-glucans from different sources with various molecular weight. Bioactive Carbohydrates and Dietary Fibre. 2014; 3(1): 11–16. 10.1016/j.bcdf.2013.12.001

[34] Gulluce M, Sahin F, Sokmen M, Ozer H, Daferera D, Sokmen A, et al Antimicrobial and antioxidant properties of the essential oils and methanol extract from *Mentha longifolia* L. ssp. Longifolia. Food Chemistry. 2007; 103(4): 1449–1456. 10.1016/j.foodchem.2006.10.061

[35] Bouaziz M, Yangui T, Sayadi S, Dhouib A. Disinfectant properties of essential oils from *Salvia officinalis* L. cultivated in Tunisia. Food and Chemical Toxicology. 2009; 47(11): 2755–2760. 10.1016/j.fct.2009.08.005 19682532

[36] Lemos JDA, Passos XS, Fernandes ODF, Paula JRD, Ferri PH, Souza LK, et al Antifungal activity from *Ocimum gratissimum* L. towards Cryptococcus neoformans. Memórias do Instituto Oswaldo Cruz. 2005;100(1): 55–58. 10.1590/S0074-02762005000100011 15867965

[37] Han QX, Chen JL, Liu BR, Tang HX. Toxic and repellent effects of several Chinese medicinal materials on the adults of Triblium castaneum (Coleoptera: Tenebrionidae). Plant Protection. 2003; 30(2): 60–63.

[38] Martins N, Barros L, Ferreira ICFR. In vivo antioxidant activity of phenolic compounds: Facts and gaps. Trends in Food Science & Technology. 2016; 48: 1–12. 10.1016/j.tifs.2015.11.008

[39] Wang LF, Chen C, Su AX, Zhang YY, Yuan J, Ju XJ. Structural characterization of phenolic compounds and antioxidant activity of the phenolic-rich fraction from defatted adlay(*Coix lachrymal-jobi* L. var. ma-yuen Stapf) seed meal. Food chemistry. 2016; 196: 509–517. 10.1016/j.foodchem.2015.09.083 26593521

[40] Silva EM, Rogez H, Larondelle Y. Optimization of extraction of phenolics from *Inga edulis* leaves using response surface methodology. Separation and Purification Technology. 2007;55(3): 381–387. 10.1016/j.seppur.2007.01.008

[41] Singh M, Kaur M, Silakari O. Flavones: An important scaffold for medicinal chemistry. European Journal of Medicinal Chemistry. 2014; 84: 206–239. 10.1016/j.ejmech.2014.07.013 25019478

[42] Mokrani A, Madani K. Effect of solvent, time and temperature on the extraction of phenolic compounds and antioxidant capacity of peach (*Prunus persica* L.) fruit. Separation and Purification Technology. 2016; 162: 68–76. 10.1016/j.seppur.2016.01.043

[43] Naczk M, Shahidi F. Phenolics in cereals, fruits and vegetables: Occurrence, extraction and analysis. Journal of Pharmaceutical and Biomedical Analysis. 2006; 41(5): 1523–1542. 10.1016/j.jpba.2006.04.002. 16753277

[44] Garcia-Salas P, Morales-Soto A, Segura-Carretero A, Fernández-Gutiérrez A. Phenolic-compound-extraction systems for fruit and vegetable samples. Molecules. 2010;15(12):8813–8826. 10.3390/molecules15128813 21131901 PMC6259353

[45] Xu GH, Ye XQ, Chen JC, Liu DH. Effect of heat treatment on the phenolic compounds and antioxidant capacity of *citrus peel*. Extract. Journal of Agricultural and Food chemistry. 2007;55: 330–335. 10.1021/jf062517l 17227062

[46] Lee LS, Choi EJ, Kim CH, Sung JM, Kim YB, Seo DH, et al Contribution of flavonoids to the antioxidant properties of common and tartary buckwheat. Journal of Cereal Science. 2016; 68: 181–186. 10.1016/j.jcs.2015.07.005

[47] Tchabo W, Ma Y, Engmann FN, Zhang H. Ultrasound-assisted enzymatic extraction (UAEE) of phytochemical compounds from mulberry (*Morus nigra*) must and optimization study using response surface methodology. Industrial Crops and Products. 2015; 63: 214–225. 10.1016/j.indcrop.2014.09.053

[48] Holetz FB, Ueda-Nakamura T, Filho BPD, Cortez DAG, Morgado-Diaz JA, Nakamura CV. Effect of essential oil of Ocimum gratissimum on the trypanosomatid Herpetomonas samuelpessoai. Acta Protozoologica. 2003; 42(4): 269–276. 10.13140/RG.2.1.1605.5208

[49] Saurav K, Kannabiran K. Cytotoxicity and antioxidant activity of 5-(2, 4-dimethylbenzyl) pyrrolidin-2-one extracted from marine Streptomyces VITSVK5 spp. Saudi journal of biological sciences. 2012; 19(1), 81–86. 10.1016/j.sjbs.2011.07.003 23961165 PMC3730557

[50] Halake K, Birajdar M, Lee J. Structural implications of polyphenolic antioxidants. Journal of Industrial and Engineering Chemistry. 2016; 35, 1–7. 10.1016/j.jiec.2016.01.003

[51] Kumarappan CT, Thilagam E, Mandal SC. Antioxidant activity of polyphenolic extracts of Ichnocarpus frutescens. Saudi journal of biological sciences. 2012; 19(3), 349–355. 10.1016/j.sjbs.2012.04.004 23961196 PMC3730722

