# Supplementary figures and images for: Optimization of Extraction Conditions for Maximal Phenolic, Flavonoid and Antioxidant Activity from Melaleuca bracteata Leaves Using the Response Surface Methodology
Source: PLoS One. 2016 Sep 9;11(9):e0162139. doi: 10.1371/journal.pone.0162139 (PMC5017642; doi:10.1371/journal.pone.0162139)

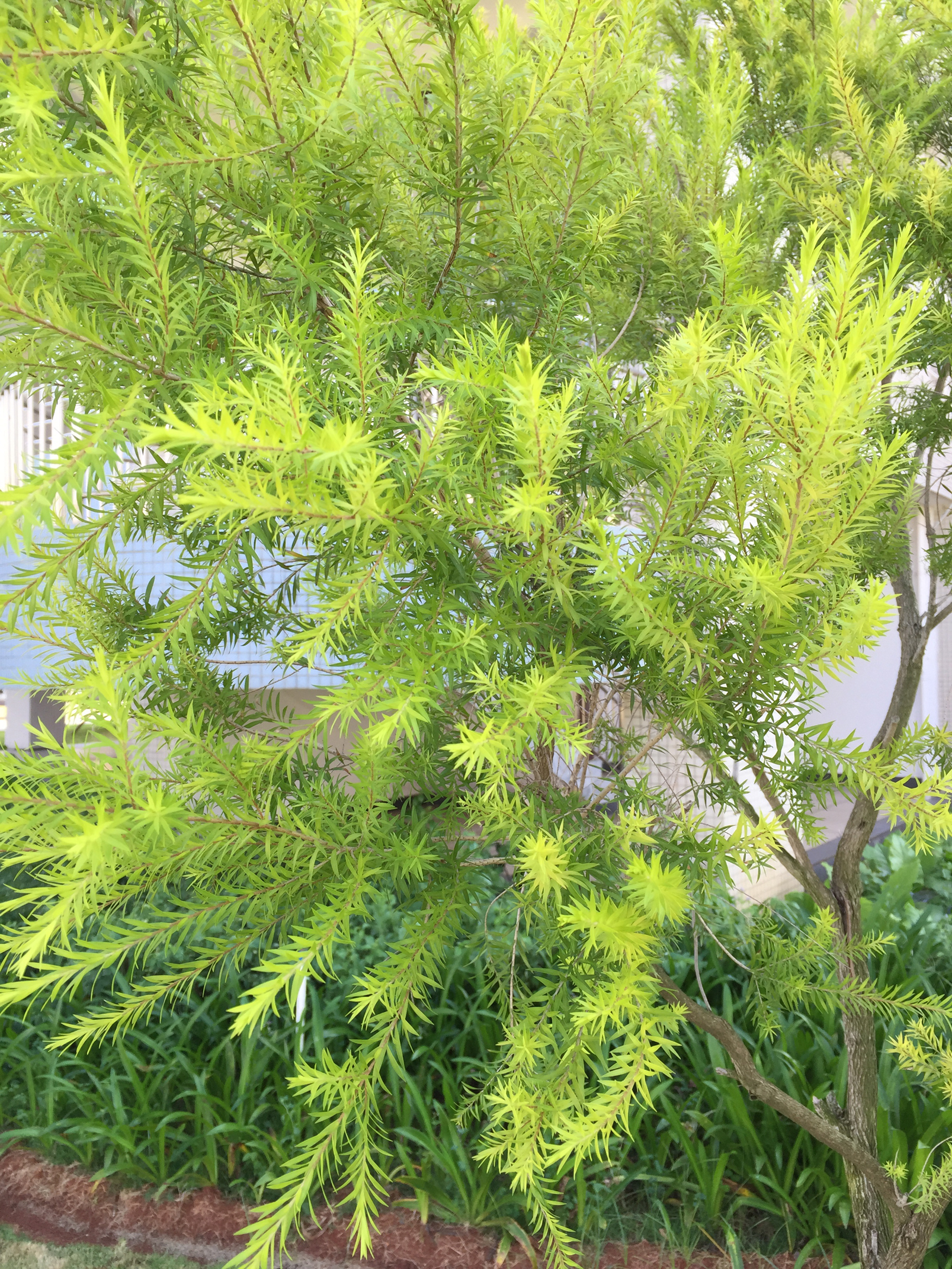

Supplement: S1 Fig — (TIF) [file pone.0162139.s001.tif]
