# Supplementary material for: Optimization of Extraction Conditions for Maximal Phenolic, Flavonoid and Antioxidant Activity from Melaleuca bracteata Leaves Using the Response Surface Methodology
Source: PLoS One. 2016 Sep 9;11(9):e0162139. doi: 10.1371/journal.pone.0162139 (PMC5017642; doi:10.1371/journal.pone.0162139)
Supplement: S1 File — (PDF) [file pone.0162139.s002.pdf]

Dear Editor:

Thank you for your hard work. The plant of *Melaleuca bracteata* used in this experiment is located in *Hainan Provincial Forestry Science Institute, Tongshen Branch Offices* (Wuzhishan city, Hainan province, China (N 109° 32′ 28″, E 18° 46′ 10″)), it is not in a national park or other protected area of land or sea, also, it is not a protected wildlife. This plant is an ordinary garden afforesting plant due to the golden yellow leaves. We reproduce this plant in order to grow at the edge of the streets or in the gardens. We agree Prof. Luo to use this plant, and hope they find valuable research result.

Best regards.

Zhang Wei

Hainan Provincial Forestry Science Institute, Tongshen Branch Offices

2016.8.5
